# Hypertension and cognitive dysfunction in elderly: blood pressure management for this global burden

**DOI:** 10.1186/s12872-016-0386-0

**Published:** 2016-11-03

**Authors:** Marijana Tadic, Cesare Cuspidi, Dagmara Hering

**Affiliations:** 1 University Clinical Hospital Centre “Dr. Dragisa Misovic”, Heroja Milana Tepica 1, 11000 Belgrade, Serbia; 2 University of Milan-Bicocca and Istituto Auxologico Italiano, Clinical Research Unit, Viale della Resistenza 23, 20036 Meda, Italy; 3 Dobney Hypertension Centre, School of Medicine and Pharmacology-Royal Perth, Hospital Unit, The University of Western Australia, Rear 50 Murray Street, 6000 Perth, Australia

**Keywords:** Arterial hypertension, Cognitive decline, Dementia, Antihypertensive therapy

## Abstract

Arterial hypertension and stroke are strong independent risk factors for the development of cognitive impairment and dementia. Persistently elevated blood pressure (BP) is known to impair cognitive function, however onset of new cognitive decline is common following a large and multiple mini strokes. Among various forms of dementia the most prevalent include Alzheimer’s disease (AD) and vascular dementia (VaD) which often present with similar clinical symptoms and challenging diagnosis. While hypertension is the most important modifiable vascular risk factor with antihypertensive therapy reducing the risk of stroke and potentially slowing cognitive decline, optimal BP levels for maintaining an ideal age-related mental performance are yet to be established. Cognition has improved following the use of at least one representative agent of the major drug classes with further neuroprotection with renin angiotensin inhibitors and calcium channel blockers in the hypertensive elderly. However, a reduction in BP may worsen cerebral perfusion causing an increased risk of CV complications due to the J-curve phenomenon. Given the uncertainties and conflicting results from randomized trials regarding the hypertension management in the elderly, particularly octogenarians, antihypertensive approaches are primarily based on expert opinion. Herein, we summarize available data linking arterial hypertension to cognitive decline and antihypertensive approach with potential benefits in improving cognitive function in elderly hypertensive patients.

## Background

The relationship between high BP and cognitive dysfunction has generated great interest and broad investigation over the past few decades [1, 2]. While concerns have raised over cerebral perfusion, blood flow and BP levels in the elderly, particularly following a stroke, there is limited evidence whether the prevention of dementia or slowing cognitive decline is associated with the BP reduction per se or specific drug properties [3–5]. Dementia represents one of the major and growing global public health problems affecting approximately 47.5 million people worldwide with 7.7 million new diagnosed cases every year (http://www.who.int/mediacentre/factsheets/fs362/en/). It has been estimated that the annual global dementia-related healthcare cost was 604 billion dollars in 2010 [6]. AD contributes to approximately 70 % of all cases followed by VaD accounting for nearly 25–30 %. With prolonged life expectancy and the growing prevalence of uncontrolled hypertension, the worldwide incidence of patients with dementia is expected to double by 2030 reaching 75.6 million and rise even further to 135.5 million by 2050 [5, 7]. Given the link between elevated BP and cognitive impairment, an improvement in BP control may reduce the risk attributable to dementia, its progression over time and possibly improve patient outcomes. Limited evidence also exists regarding chronic kidney disease, small vessel disease and other modifiable risk factors with a specific focus on midlife hypertension, midlife obesity, midlife dyslipidemia or depression to prevent future cognition impairment.

### Hypertension and cognitive dysfunction related with stroke

The association between hypertension and stroke-related dementia is well recognized. Evidence from numerous studies indicates the development of cognitive decline following stroke. Dementia has been reported in approximately 10–30 % of patients 3 months after stroke [8, 9]. A meta-analysis of 7511 patients indicated that 10 % of patients developed dementia before to first stroke, 10 % had new dementia directly after first stroke, and >30 % of patients had dementia after recurrent stroke [9]. The risk of dementia increased two to five times following stroke indicating it is a critical contributor in this scenario [8–11]. The prevalence of cognitive decline following stroke has been shown to remain persistently high. An observational longitudinal study of 4212 post stroke patients revealed an occurrence of cognitive impairment in 22 % at 3 months, 22 % at 5 years and 21 % at 14 years of follow-up [12] with cognitive impairment in some patients detected within 7 days which remained stable 3 months after stroke.

The interaction between brain infarction and the risk of clinical dementia has been reported in the Nun study [13]. In this autopsy research, patients with neurological features of AD and ≥1 lacunar stroke in the thalamus, basal ganglia, or deep white matter had a 20 times higher risk of clinical dementia comparing to AD patients without infarcts [13]. Although arterial hypertension plays a causative role in cerebral small vessel disease including lacunar infarcts [14], further studies need to determine whether maintaining BP control may prevent from lacunar infarcts and associated cognitive dysfunction and dementia.

### Hypertension and cognitive dysfunction unrelated to stroke

The relationship between increased BP and cognitive dysfunction in the absence of stroke is controversial and not completely understood. Numerous studies have demonstrated an inverse association between BP levels and cognitive function. The Framingham Heart Study has showed that cognitive function was associated with the basic BP measurement [15]. Among 1702 subjects, cognitive performance worsened over a 12 to 14-year period and was negatively associated with initial systolic (S) and diastolic (D) BP levels. Another study of a total of 3735 male subjects indicated that midlife elevated SBP predicted a future reduction in cognitive function [16]. There was an increased risk for intermediate and poor cognitive function with every increment increase in SBP by 10 mmHg irrespective of the history of stroke, coronary heart disease and subclinical atherosclerosis indicating that BP control in early life may reduce the risk for cognitive impairment in old age [16]. A further observation was derived from a study of 2068 individuals ≥65 years demonstrating a U-shaped association between baseline SBP and DBP and mental decline assessed after 9 years of follow-up [17]. Elevated baseline SBP of ≥160 mmHg was associated with a 14 % (95 % CI: 4–25 %) increase risk of cognitive dysfunction during 9 years of follow-up. Recently, a study of 1115 very old individuals (85 year and older) showed that cognitive dysfunction was the highest among participants with SBP >165 mmHg or <125 mmHg when compared to individuals with SBP between 126 and 139 mmHg [18] suggesting the U-curve ratio between BP and cognitive dysfunction. This could be partly explained by the fact that age at BP measurements could be related with the inconsistent associations between BP and cognitive function.

Optimal BP targets in older adults has become even more controversial following the findings from the recent longitudinal Milan Geriatrics 75+ Cohort Study [19]. In this study, 1046 out of 1587 outpatients died during 10-year follow-up and SBP/DBP of 165/85 mmHg were associated with the lowest mortality. In contrast, patients with SBP <120 mmHg and SBP between 120 and 139 (prehypertension) mmHg had 1.64-fold (95 % confidence intervals, CI 1.21–2.23) and 1.32-fold (95 % CI 1.10–1.60) higher mortality risk than patients with SBP of 160–179 mmHg (P values 0.001 and 0.004). Most interestingly, higher SBP was associated with lower mortality in patients with impaired Mini-Mental State Examination and Basic Activities of Daily Living [19]. These findings suggest that patients with impaired functional and cognitive status may benefit from higher BP levels, this however merits further clinical research.

The independent association between low daytime SBP (≤128 mmHg) and a progression of cognitive decline in patients with dementia and cognitive impairment has been recently demonstrated in another study cohort (mean age 79 ± 5), this however was not observed in subjects with low SBP without antihypertensive treatment [20].

Notably, the adverse effects of high BP on cognition have not consistently been confirmed [21, 22]. This could be partly explained by inconsistent subject and cohort selection including ethnicity and differences in methods used for the evaluation of cognitive function or other confounding factors beyond BP levels which may be additively and synergistically involved in the process of cognitive dysfunction. Considering the competing risk exists in elderly subjects, the effects between cognitive dysfunction and blood pressure may only be modest and even biased. In other words, only survivors of major adverse vascular events can provide the opportunities for investigators to observe the relationship between BP and cognition impairment.

Nevertheless, persistently elevated BP has been shown to be an important contributor to dementia [23–25]. A longitudinal study with a duration of 21 years of follow-up has supported the concept that midlife arterial hypertension (SBP ≥ 160 mmHg) or high serum cholesterol levels (≥6.5 mmol/L) significantly increase the late-life risk of AD, particularly in the combination of these abnormalities [23]. Furthermore evidence suggests that both midlife and late-life hypertension increase the risk of dementia over time [26]. The negative influence of midlife BP on dementia has also been showed in the recent Uppsala Longitudinal Study of Adult Men with 40 years of follow-up [27]. In this research, 349 out of the 2268 enrolled individuals developed dementia over the longer-term where SBP was found to be a critical contributor to the elevated risk of vascular and all-type dementia [27]. The risk was further potentiated by the presence of other CV risk factors including obesity and dyslipidemia [27] which is line with previous findings showing the influence of obesity-associated metabolic abnormalities on cognitive function [28]. Another study showed that midlife but not late-life hypertension was associated with greater cognitive decline over 20-year follow-up in the recent ARIC (Atherosclerosis Risk in Communities) neurocognitive trail [29].

However, some studies showed that high BP bore no relationship with dementia and even suggested that dementia is related to low BP due to J-curve phenomenon [30–32], particularly in the elderly and very elderly individuals [32]. While mechanisms linking low BP to cognitive dysfunction are not entirely understood, the death of neurons in deep brain structures that are responsible for BP control in addition to physical inactivity and the fall in BP common in old individuals may play a role in this scenario.

The association between BP and AD is more complex. Despite a 40 year follow-up duration, midlife BP was unrelated to the risk of AD in the Uppsala Longitudinal Study of Adult Men [27]. Similar findings have been reported in other studies [33, 34] with increased DBP associated with a significantly lower risk of AD [34].

While the role of ethnicity on cognitive decline has not yet been established, a recent meta-analysis that included 28,477 participants revealed that hypertensive African Americans are at the highest risk of cognitive limitations (11 %, *p* = 0.01) when compared to non-hypertensive African Americans and non-African Americans [35].

Recent SPRINT trial showed that the more intensive systolic BP targets of <120 mmHg in non-diabetic subjects are superior over the standard systolic BP target of <140 mmHg [36]. In the elderly patients (>75 years), intensive treatment was more effective than standard BP lowering (HR 0.67; 95 %: 0.51–0.86) [36]. This suggests that the target systolic BP should be <120 mmHg in the elderly patients. Interestingly, the lower systolic BP target was beneficial for both the fit elderly and the frail elderly. However, the highest benefit was in elderly with average fit status. In the fit elderly hypertensive patients the HR was 0.47 (95 % CI: 0.13–1.39), less fit elderly HR 0.63 (95 % CI: 0.43–0.91) and the frail elderly HR 0.68 (95 % CI: 0.45–1.01) [36].

The results from the SPRINT MIND trial, which is part of the SPRINT study [36], will provide answers on many questions including the optimal target BP value for prevention of cognitive dysfunction. The primary objective of the SPRINT MIND trial is to determine whether intensive BP lowering with target systolic BP <120 mmHg is associated with a greater reduction in the incidence of all-cause dementia comparing with a standard treatment target of 140 mm Hg.

### Mechanisms linking blood pressure to cognitive dysfunction

The mechanisms underlying cognitive impairment are complex and not clearly understood [37]. Age-related functional and structural changes in cerebrovascular small and large blood vessels appear to play a key role in this context. A reduced number of cerebral cortical capillaries with thickening and fibrotic basement membranes in addition to changes in white matter have been found in the elderly [38, 39]. The abnormalities associated with aging adversely impact brain circulation causing a reduction in resting cerebral blood flow with subsequent dysfunction of regulatory cerebral mechanisms [40] which are further potentiated by arterial hypertension. Subsequently, hypertension-related cognitive dysfunction develops as a result of the imbalance in autoregulation of cerebral blood flow and cerebral vascular alterations.

In addition, arterial stiffness may explain the association between hypertension and cognitive dysfunction [41] and provide explanations for the conflicting observations. Namely, the authors stated that increased arterial stiffness is better predictor of cognitive decline than BP level and proposed that arterial stiffness might be a target in the prevention of cognitive decline in hypertensive patients. Cooper et al. reported that relationship between aortic stiffness and cognitive function in older adults is the consequence of damage in cerebral microvascularisation and microvascular parenchymal injury [42, 43].

A recent study in elderly subjects has demonstrated a decreased temporal and occipital brain perfusion, total and regional cortical thickness in participants with hypertension when compared to those without hypertension [44]. The negative impact of hypertension on total brain perfusion remained reduced irrespective of patients’ age. Reduced total brain perfusion predicted a decrease in cortical thickness whereas antihypertensive therapy was unrelated to total cerebral perfusion or cortical thickness [44].

Brain regions which are particularly vulnerable to alterations with advanced age and increased BP involve the prefrontal cortex, hippocampus, inferior temporal cortex and inferior parietal lobule [40, 45–47]. This observation indicates that high BP alters grey matter structure by age-independent mechanisms.

Changes in white matter commonly occur in the elderly, however the mechanisms underlying their decrease are not fully understood. Cerebral small vessel disease importantly contributes to a reduction in white matter [48] which has been shown to be associated with an increased risk of stroke, cognitive dysfunction, dementia and death [49]. Hypertension per se plays a causative role in the development of cerebral small vessel disease resulting in lacunar infarcts [50].

Large artery remodeling is another potential mechanism related with cognitive decline. Increased pulse pressure and arterial stiffness which are a hallmark of hypertension have been linked to white matter degradation, cognitive dysfunction and dementia [51, 52]. Hypertension-related cerebral white matter degradation has been particularly found in the frontal lobe [49].

Additionally, accumulation of beta amyloid protein in the brain related with high BP could be a potential mechanism that connects BP level and cognitive dysfunction [53].

Activation of the renin-angiotensin-aldosterone system and oxidative stress are further mechanisms implicated in hypertension-related cognitive dysfunction [54, 55]. Endothelial dysfunction which is a common feature of hypertension leads to endothelium-mediated constriction and brain damage [56]. The alterations in vasodilator mechanisms including nitric oxide, epoxyeicosatrienoic acids and ion channels adversely impact endothelial function [54]. Hypertension induces blood–brain barrier damage through direct effect on oxidative stress, inflammation and vasoactive substances [54]. Consequently, neurons are more vulnerable to cytotoxic molecules leading to neuronal loss, white matter degradation, cognitive decline and impaired recovery following cerebral ischemia [54].

### Antihypertensive therapy and cognitive function

While several well-designed randomized controlled trials reported the favorable influence of antihypertensive therapy in the prevention or slowing of cognitive deterioration, its preventive efficacy disputable.

A meta-analysis that included 15,936 hypertensive subjects with average age of 75.4 years and BP levels of 171/86 mmHg across the studies failed to confirm that lowering BP in late-life prevents the development of dementia or cognitive impairment in hypertensive subjects without a history of cerebrovascular disease [57]. On the contrary, the well-known PROGRESS (Perindopril Protection Against Recurrent Stroke Study) [58] and Syst-Eur (Systolic Hypertension in Europe) trials [59] have demonstrated a significant reduction in stroke-related cognitive decline. The interest for the effect of BP reduction on cognitive decline in middle-aged hypertensive patients is increasing [60]. The Syst-Eur study reported a 50 % reduction in incident dementia during 2-year follow-up period in subjects older than 60 years [59]. Hypertensive middle-aged patients should be routinely tested for cognitive dysfunction because it may be an early predictor of dementia and antihypertensive treatment could slow cognitive decline in this patients.

More recently, another interesting observation comes from a meta-analysis comparing the effectiveness of different antihypertensive drug classes on the incidence of dementia and cognitive function [61]. Antihypertensive therapy was associated with favorable effects on cognitive function [effect size 0.05, 95 % confidence interval: 0.02–0.07] irrespective of drug classes. The risk of all-cause dementia was reduced by 9 % following BP lowering therapy when compared to the control group (hazard ratio 0.91, 95 % CI: 0.89–0.94) [61]. While no significant changes in average BP levels were noted across the various antihypertensive drug classes, angiotensin II receptor blockers (ARB) were superior to placebo in improving overall cognition and were more effective than β-blockers, diuretics and angiotensin-converting enzyme inhibitors (ACEI) [61].

Several reasons have been suggested for the different cognitive function responses to antihypertensive medication and a resultant lack of coherent recommendation to support the use of any specific drug class in the prevention or slowing cognitive decline [62]. No previous studies have been designed specifically to focus on cognition, instead were concentrated on elderly populations with low risk or minimal baseline cognitive impairment and/or white matter lesions with no long-term follow-up.

Studies regarding the influence of different antihypertensive agents on cognitive function were mainly focused on the effect of the blockers of the renin-angiotensin-aldosterone (RAAS) and calcium channel blockers. These investigations showed the favorable effect of RAAS and calcium channel blockers on mental status, which seems to be more pronounced than in other antihypertensive groups. However, we need randomized trials that would provide answer on this important question. Possibly substudies of the SPRINT study will give satisfactory response.

### Renin-angiotensin-aldosterone system antagonists

The activity of the RAAS system has been shown to be implicated in cognitive decline and dementia. Indeed, higher levels of circulating aldosterone were related with impaired cerebral function in older hypertensive patients [55]. Activation of angiotensin 1 receptor is associated with inflammation, blood–brain barrier damage and a decrease in blood brain flow. In this context, an inhibition of the RAAS in the hippocampus (which plays a significant part in cognition) using blood–brain barrier-penetrating RAAS inhibitors may have favorable effects in preventing cognitive decline [63]. Therapy with ARBs and ACEIs, particularly using brain-penetrating drugs such as captopril, perindopril and telmisartan appears to improve blood–brain barrier function, increase cerebral blood flow and reduce inflammation.

#### Angiotensin-converting enzyme inhibitors

ACEIs have been found to reduce the accumulation of amyloid β (Aβ) peptides through substance P that potentially may degrade brain Aβ peptide and increase the acetylcholine [64]. This drug class can also decrease neuronal injury by an anti-oxidative pathway. Given that ACE has been found to be overexpressed in the hippocampus, frontal cortex, and caudate nucleus in patients with AD [65], ACEI could be of clinical benefits in this patient cohort.

The HOPE (Heart Outcomes Prevention Evaluation) trial followed over 4.5 years 9297 patients with vascular diseases and demonstrated a 41 % decrease in stroke-related cognitive decline following therapy with ramipril when compared to placebo [66]. Although a relatively modest reduction in SBP/DBP (3.8/2.8 mmHg) levels was achieved, the positive effect was especially evident in regards to cognition and language disorders.

In the PROGRESS trial which involved 6105 patients with cerebrovascular disease, treatment with perindopril and indapamide for 4 years reduced the relative risk of cognitive decline by 19 % (95 % CI: 4 to 32 %) in the total population and 45 % in patients with recurrent strokes (95 % CI: 21 to 61 %) [58]. In the total population the risk of dementia also decreased by 12 % (95 % CI: −8 to 28 %) and by 34 % in patients with recurrent strokes (95 % CI: 3 to 55 %). These beneficial effects of perindopril and indapamide therapy on dementia and cognition were noticed irrespective of the history of hypertension including both hypertensive and non-hypertensive patients. A significant reduction in the risk of dementia (by 23 %; 95 % CI: 0 to 41 %) was achieved with dual therapy accompanied by a substantial reduction in BP (−12/5 mmHg for SBP/DBP) but not monotherapy (−8 %; 95 % CI = −48 to 21 %) resulting in a lower BP reduction (−5/3 mmHg for SBP/DBP) [58].

Another interesting observation from a randomized study has demonstrated that ACEIs which specifically penetrate the blood–brain barrier (perindopril, captopril) may be more effective in slowing cognitive decline in mild to moderate AD patients when compared to non-brain-penetrating ACEIs or calcium channel blockers [67].

#### Angiotensin II receptor blocker

While ARBs may have a neuroprotective effect via crossing the blood–brain barrier and increasing brain blood flow, not all studies have proven this benefit. The SCOPE (Study on Cognition and Prognosis in the Elderly) trial included 4964 hypertensives aged 70–89 with SBP and DBP of 160–170/90–99 mmHg followed over 3.97 years [68]. In this study, the effect of candesartan on dementia and/or cognitive decline was not dissimilar to that of placebo [68].

Another prospective study of 819,491 male participants (98 %) aged ≥65 has shown that time to incident and deterioration of AD or dementia were significantly reduced with ARBs comparing to ACEIs or other CV drugs [69].

Further supportive evidence for the use of ARBs in cognitive decline comes from a randomized study of 160 patients (76 men, 84 women) aged 61–75 years which compared the effects of telmisartan/hydrochlorothiazide (HTZ) to lisinopril/HTZ on cognitive function and ambulatory BP levels [70]. The greater BP lowering effects achieved with the telmisartan/HTZ combination was accompanied by an improvement in some of the components of cognitive function, particularly episodic memory and visuospatial abilities at both 12 and 24 weeks of follow-up. In contrast, no significant changes in any of the cognitive function test scores were observed following treatment with lisinopril/HCTZ at any time of the study [70].

Finally, a large meta-analysis that included 19 randomized trials (*n* = 18,515) and 11 studies (*n* = 831,674) of patients with hypertension without previous cerebrovascular disorders demonstrated that antihypertensive treatment has favorable effects on cognitive decline, however this benefit may differ between drug classes with the most pronounced effect achieved with ARBs [61]. In this context, further longer-term studies are needed to determine the effectiveness of ARBs in reducing the incidence of dementia.

#### Aldosterone antagonists

Very recently, the association of aldosterone and renin levels with cognitive and cerebrovascular measures was demonstrated in a cohort of 47 subjects with mean age of 71 who were followed over a 1 year of antihypertensive therapy [55]. While higher levels of aldosterone at baseline were associated with decreased cerebrovascular function in hypertension, these effects were unrelated to the type of antihypertensive therapy or the degree of changes in aldosterone levels. Unlike aldosterone, levels of plasma renin activity bore no association with cognitive measures [55].

In line with this observation, increased plasma aldosterone concentration has been linked to impaired cognitive function in hypertensive patients [71]. Although only shown in a small number of patients (*n* = 7), therapy with aldosterone antagonists (i.e. spironolactone and eplerenone) has led to an improvement in the mini-mental state examination in treated patients when compared to controls [71].

The relationship between the protective effects of using BP lowering drugs and risk of AD was investigated in the elderly participants of the Cache County Study [72]. Although the use of any antihypertensive medication reduced the incidence of AD, the greatest effect was observed using potassium-sparing diuretics with more than a 70 % reduction in risk of AD [72].

While these findings are promising, the role that excessive aldosterone release on neurocognitive function merits further investigation. Likewise, whether mineral corticoid receptor blockade may improve cognitive function beyond CV protection needs to be confirmed in larger randomized clinical trials.

### Calcium channel blockers

Intracellular calcium homeostasis is another potential mechanism involved in cognitive function. Evidence suggests that aging impairs ability of the brain intracellular calcium degradation which is likely to induce cellular damage leading to neural death and resultant cognitive dysfunction. Indeed, 3 months therapy with nimodipine commencing 7–14 days after cerebral infarction resulted in memory improvement [73]. Further supportive findings for the neuroprotective effects of calcium antagonists have been demonstrated in 1241 elderly hypertensive patients with memory impairment [74]. The use of calcium antagonists decreased the risk of cognitive impairment and AD independently of BP levels when compared to patients not receiving calcium blockers [74]. The long-term effects of antihypertensive therapy initiated with a long-acting dihydropinidine (nitrendypine) has been demonstrated in the double-blind, placebo-controlled Syst-Eur trail in which the incidence of dementia was reduced by 55 % [59].

### Diuretics

Determining the exact effects of diuretics on cognitive function remain challenging as most previous studies applied them as concomitant antihypertensive agents rather than monotherapy.

In the Syst-Eur trial HTZ was added to nitrendipine and/or ACEI (enalapril) and indicated a reduced risk of dementia following the long-term therapy [59]. The PROGRESS study used indapamide in combination with perindopril with a resulting decrease in the risk of dementia and cognitive decline after recurrent stroke when compared to perindopril alone or placebo [58]. However, the Systolic Hypertension in the Elderly Program trial did not find difference in the occurrence of cognitive dysfunction in patients taking active treatment (low-dose diuretic and/or beta blockers) or placebo [75].

### Beta-adrenergic blocking agents

The influence of β-blockers with their potential beneficial effects on cognitive function has not yet been clarified with conflicting findings reported from different studies.

One of the major limitations of previous studies is that β-blockers are not commonly used as monotherapy in the elderly but often in combination with ACEI/ARB, calcium channel blockers and/or diuretics.

Available evidence suggests that a reduction in the incidence of AD in participants with normal cognition appears to be achieved with all antihypertensive drug classes including β-blockers [76]. However, the neuroprotective effects of β-blockers seemed to be inferior when compared to other classes [76].

On the contrary, the Honolulu-Asia Aging Study which included 2197 hypertensive males (mean age 77 years) has found that the use of β-blockers alone was related with a decreased risk of cognitive declining [77]. The efficacy of β-blockers in slowing cognitive impairment was more pronounced in men with diabetes and those older than 75 years and with pulse pressure ≥70 mm Hg. In contrast, the use of diuretics, calcium channel blockers, ACEIs or vasodilators alone failed to demonstrate any cognitive improvement [77]. However, this was not confirmed by another cognitive randomized placebo-controlled sub-study which demonstrated no difference between the initiation of treatment with β-blocker, diuretic or placebo on the change of cognitive function over 54 months in hypertensive older adults [78]. Whether differences in the efficacy of β-blockers on cognitive function are related to specific drug properties, selectivity, pharmacokinetics or study cohorts requires further investigation.

## Conclusion

While the contribution of hypertension to cognitive dysfunction and dementia is well-recognized, the use of antihypertensive approaches for the prevention of cognitive decline continues to be debated. In view of the global burden of hypertension, the aging population, the low quality of life associated with cognitive decline and/or dementia resulting in increasing costs to health care systems, there is rationale for improving BP control. Special attention should focused on the treatment of midlife hypertension which is likely to reduce the incidence of dementia in late-life which is beyond cardiac, renal and vascular protection. Evidence supports the use of any BP lowering drugs with greater efficacy leaning to treatment with ARBs, ACEIs, calcium channel blockers and aldosterone antagonists for the prevention of cognitive decline in hypertensive patients. Combination of two or more antihypertensive agents is preferable to monotherapy due to additive and synergistic effects on BP control, prevention of stroke and possibly cognitive impairment. Notably, cautious hypertension management is required in the elderly and very old frail subjects as per the recent expert opinion recommendations [79]. Given that there is potential for (1) increasing incidence of dementia and mortality in old elderly with low BP achieved with antihypertensive drug therapy and (2) worsening further prognosis in hypertensive patients with existing impaired cognition, a therapeutic approach in this cohort needs to be established. Cognitive tests should be implemented into clinical practice, particularly in the old hypertensives prior to commencing therapy.

## Abbreviations

ACEI: Angiotensin converting enzyme inhibitor
AD: Alzheimer’s disease
ARB: Angiotensin II receptor blocker
BP: Blood pressure
CI: Confidence interval
CV: Cardiovascular
DBP: Diastolic blood pressure
RAAS: Renin-angiotensin-aldosterone system
SBP: Systolic blood pressure
VaD: Vascular dementia

## References

[1] Shapiro AP, Miller RE, King E, Gincherreau EH, Fitzgibbon K (1982). Behavioral consequences of mild hypertension. Hypertension.

[2] Scherr PA, Hebert LE, Smith LA, Evans DA (1991). Relation of blood pressure to cognitive function in the elderly. Am J Epidemiol.

[3] Plassman BL, Williams JW, Burke JR, Holsinger T, Benjamin S (2010). Systematic review: factors associated with risk for and possible prevention of cognitive decline in later life. Ann Intern Med.

[4] Daviglus ML, Bell CC, Berrettini W, Bowen PE, Connolly ES, Cox NJ (2010). National Institutes of Health state-of-the-science conference statement: preventing Alzheimer disease and cognitive decline. Ann Intern Med.

[5] Gorelick PB, Nyenhuis D, Materson BJ, Calhoun DA, Elliott WJ, Phillips RA, Taler SJ, Townsend RR, American Society of Hypertension Writing Group (2012). Blood pressure and treatment of persons with hypertension as it relates to cognitive outcomes including executive function. J Am Soc Hypertens.

[6] Wimo A, Jönsson L, Bond J, Prince M, Winblad B, Alzheimer Disease International (2013). The worldwide economic impact of dementia 2010. Alzheimers Dement.

[7] Hebert LE, Beckett LA, Scherr PA, Evans DA (2001). Annual incidence of Alzheimer disease in the United States projected to the years 2000 through 2050. Alzheimer Dis Assoc Disord.

[8] Tatemichi TK, Desmond DW, Mayeux R, Paik M, Stern Y, Sano M, Remien RH, Williams JB, Mohr JP, Hauser WA (1992). Dementia after stroke: baseline frequency, risks, and clinical features in a hospitalized cohort. Neurology.

[9] Pendlebury ST, Rothwell PM (2009). Prevalence, incidence, and factors associated with pre-stroke and post-stroke dementia: a systematic review and meta-analysis. Lancet Neurol.

[10] Prencipe M, Ferretti C, Casini AR, Santini M, Giubilei F, Culasso F (1997). Stroke, disability, and dementia: results of a population survey. Stroke.

[11] Linden T, Skoog I, Fagerberg B, Steen B, Blomstrand C (2004). Cognitive impairment and dementia 20 months after stroke. Neuroepidemiology.

[12] Douiri A, Rudd AG, Wolfe CD (2013). Prevalence of post-stroke cognitive impairment: South London Stroke Register 1995–2010. Stroke.

[13] Snowdon DA, Greiner LH, Mortimer JA, Riley KP, Greiner PA, Markesbery WR (1997). Brain infarction and the clinical expression of Alzheimer disease: the Nun study. JAMA.

[14] Tsai CF, Anderson N, Thomas B, Sudlow CL (2015). Risk factors for ischemic stroke and its subtypes in Chinese vs. Caucasians: systematic review and meta-analysis. Int J Stroke.

[15] Elias MF, Wolf PA, D’Agostino RB, Cobb J, White LR (1993). Untreated blood pressure level is inversely related to cognitive functioning: the Framingham study. Am J Epidemiol.

[16] Launer LJ, Masaki K, Petrovitch H, Foley D, Havlik RJ (1995). The association between midlife blood pressure levels and late-life cognitive function. The Honolulu-Asia aging study. JAMA.

[17] Glynn RJ, Beckett LA, Hebert LE, Morris MC, Scherr PA, Evans DA (1999). Current and remote blood pressure and cognitive decline. JAMA.

[18] Weidung B, Littbrand H, Nordström P, Carlberg B, Gustafson Y (2016). The association between SBP and mortality risk differs with level of cognitive function in very old individuals. J Hypertens.

[19] Ogliari G, Westendorp RG, Muller M, Mari D, Torresani E, Felicetta I, Lucchi T, Rossi PD, Sabayan B, de Craen AJ (2015). Blood pressure and 10-year mortality risk in the Milan Geriatrics 75+ Cohort Study: role of functional and cognitive status. Age Ageing.

[20] Mossello E, Pieraccioli M, Nesti N, Bulgaresi M, Lorenzi C, Caleri V, Tonon E, Cavallini MC, Baroncini C, Di Bari M, Baldasseroni S, Cantini C, Biagini CA, Marchionni N, Ungar A (2015). Effects of low blood pressure in cognitively impaired elderly patients treated with antihypertensive drugs. JAMA Intern Med.

[21] Farmer ME, Kittner SJ, Abbott RD, Wolz MM, Wolf PA, White LR (1990). Longitudinally measured blood pressure, antihypertensive medication use, and cognitive performance: the Framingham Study. J Clin Epidemiol.

[22] Zhu L, Viitanen M, Guo ZC, Winblad B, Fratiglioni L (1998). Blood pressure reduction, cardiovascular diseases, and cognitive decline in the mini-mental state examination in a community population of normal very old people: a three-year follow-up. J Clin Epidemiol.

[23] Kivipelto M, Helkala EL, Laakso MP (2001). Midlife vascular risk factors and Alzheimer’s disease in later life: longitudinal, population based study. BMJ.

[24] Skoog I, Lernfelt B, Landahl S (1996). 15-year longitudinal study of blood pressure and dementia. Lancet.

[25] Launer LJ, Ross GW, Petrovitch H (2000). Midlife blood pressure and dementia: the Honolulu-Asia aging study. Neurobiol Aging.

[26] Ninomiya T, Ohara T, Hirakawa Y, Yoshida D, Doi Y, Hata J, Kanba S, Iwaki T, Kiyohara Y (2011). Midlife and late-life blood pressure and dementia in Japanese elderly: the Hisayama study. Hypertension.

[27] Ronnemaa E, Zethelius B, Lannfelt L, Kilander L (2011). Vascular risk factors and dementia: 40-year follow-up of a populationbased cohort. Dement Geriatr Cogn Disord.

[28] Laitala VS, Kaprio J, Koskenvuo M, Raiha I, Rinne JO, Silventoinen K (2011). Association and causal relationship of midlife obesity and related metabolic disorders with old age cognition. Curr Alzheimer Res.

[29] Gottesman RF, Schneider AL, Albert M, Alonso A, Bandeen-Roche K, Coker L, Coresh J, Knopman D, Power MC, Rawlings A, Sharrett AR, Wruck LM, Mosley TH (2014). Midlife hypertension and 20-year cognitive change: the atherosclerosis risk in communities neurocognitive study. JAMA Neurol.

[30] Morris MC, Scherr PA, Hebert LE, Glynn RJ, Bennett DA, Evans DA (2001). Association of incident Alzheimer disease and blood pressure measured from 13 years before to 2 years after diagnosis in a large community study. Arch Neurol.

[31] Verghese J, Lipton RB, Hall CB, Kuslansky G, Katz MJ (2003). Low blood pressure the risk of dementia in very old individuals. Neurology.

[32] Molander L, Gustafson Y, Lovheim H (2010). Longitudinal associations between blood pressure and dementia in the very old. Dement Geriatr Cogn Disord.

[33] Oveisgharan S, Hachinski V (2010). Hypertension, executive dysfunction, and progression to dementia: the canadian study of health and aging. Arch Neurol.

[34] Yang YH, Roe CM, Morris JC (2011). Relationship between late-life hypertension, blood pressure, and Alzheimer’s disease. Am J Alzheimers Dis Other Demen.

[35] Hajjar I, Wharton W, Mack WJ, Levey AI, Goldstein FC (2016). Racial disparity in cognitive and functional disability in hypertension and all-cause mortality. Am J Hypertens.

[36] Wright JT, Williamson JD, Whelton PK, Snyder JK, Sink KM, Rocco MV, Reboussin DM, Rahman M, Oparil S, Lewis CE, Kimmel PL, Johnson KC, Goff DC, Fine LJ, Cutler JA, Cushman WC, Cheung AK, Ambrosius WT, SPRINT Research Group (2015). A randomized trial of intensive versus standard blood-pressure control. N Engl J Med.

[37] Iadecola C (2014). Hypertension and dementia. Hypertension.

[38] Farkas E, Luiten PG (2001). Cerebral microvascular pathology in aging and Alzheimer’s disease. Prog Neurobiol.

[39] Kalaria RN (2009). Linking cerebrovascular defense mechanisms in brain ageing and Alzheimer’s disease. Neurobiol Aging.

[40] Gąsecki D, Kwarciany M, Nyka W, Narkiewicz K (2013). Hypertension, brain damage and cognitive decline. Curr Hypertens Rep.

[41] Hajjar I, Goldstein FC, Martin GS, Quyyumi AA (2016). Roles of arterial stiffness and blood pressure in hypertension-associated cognitive decline in healthy adults. Hypertension.

[42] Cooper LL, Woodard T, Sigurdsson S, van Buchem MA, Torjesen AA, Inker LA, Aspelund T, Eiriksdottir G, Harris TB, Gudnason V, Launer LJ, Mitchell GF (2016). Cerebrovascular damage mediates relations between aortic stiffness and memory. Hypertension.

[43] Cooper LL, Mitchell GF (2016). Aortic stiffness, cerebrovascular dysfunction, and memory. Pulse (Basel).

[44] Alosco ML, Gunstad J, Xu X, Clark US, Labbe DR, Riskin-Jones HH, Terrero G, Schwarz NF, Walsh EG, Poppas A, Cohen RA, Sweet LH (2014). The impact of hypertension on cerebral perfusion and cortical thickness in older adults. J Am Soc Hypertens.

[45] Raz N, Rodrigue KM, Haacke EM (2007). Brain aging and its modifiers: insights from in vivo neuromorphometry and susceptibility weighted imaging. Ann N Y Acad Sci.

[46] Raz N, Rodrigue KM, Kennedy KM, Acker JD (2007). Vascular health and longitudinal changes in brain and cognition in middle-aged and older adults. Neuropsychology.

[47] Debette S, Markus HS (2010). The clinical importance of white matter hyperintensities on brain magnetic resonance imaging: systematic review and meta-analysis. BMJ.

[48] Pantoni L (2010). Cerebral small vessel disease: from pathogenesis and clinical characteristics to therapeutic challenges. Lancet Neurol.

[49] Raz N, Yang YQ, Rodrigue KM, Kennedy KM, Lindenberger U, Ghisletta P (2012). White matter deterioration in 15 months: latent growth curve models in healthy adults. Neurobiol Aging.

[50] Hering D, Piskunowicz M. Hypertension and ischaemic stroke subtypes. In: Coca A, Manica G, Rosei EA, editors. Hypertension and brain damage. European Society of Hypertension, Working Group Hypertension and the Brain; 2016. doi: 10.1007/978-3-319-32074-8

[51] Waldstein SR, Rice SC, Thayer JF, Najjar SS, Scuteri A, Zonderman AB (2008). Pulse pressure and pulse wave velocity are related to cognitive decline in the Baltimore Longitudinal Study of Aging. Hypertension.

[52] Qiu C, Winblad B, Viitanen M, Fratiglioni L (2003). Pulse pressure and risk of Alzheimer disease in persons aged 75 years and older: a community-based, longitudinal study. Stroke.

[53] Carnevale D, Mascio G, D’Andrea I, Fardella V, Bell RD, Branchi I, Pallante F, Zlokovic B, Yan SS, Lembo G (2012). Hypertension induces brain β-amyloid accumulation, cognitive impairment, and memory deterioration through activation of receptor for advanced glycation end products in brain vasculature. Hypertension.

[54] Pires PW, Dams Ramos CM, Matin N, Dorrance AM (2013). The effects of hypertension on the cerebral circulation. Am J Physiol Heart Circ Physiol.

[55] Hajjar I, Hart M, Mack W, Lipsitz LA (2015). Aldosterone, cognitive function, and cerebral hemodynamics in hypertension and antihypertensive therapy. Am J Hypertens.

[56] Uiterwijk R, Huijts M, Staals J, Rouhl RP, De Leeuw PW, Kroon AA, Van Oostenbrugge RJ (2016). Endothelial activation is associated with cognitive performance in patients with hypertension. Am J Hypertens.

[57] McGuinness B, Todd S, Passmore P, Bullock R (2009). Blood pressure lowering in patients without prior cerebrovascular disease for prevention of cognitive impairment and dementia. Cochrane Database Syst Rev.

[58] Tzourio C, Anderson C, Chapman N, Woodward M, Neal B, MacMahon S, Chalmers J, PROGRESS Collaborative Group (2003). Effects of blood pressure lowering with perindopril and indapamide therapy on dementia and cognitive decline in patients with cerebrovascular disease. Arch Intern Med.

[59] Forette F, Seux ML, Staessen JA, Thijs L, Babarskiene MR, Babeanu S, Bossini A, Fagard R, Gil-Extremera B, Laks T, Kobalava Z, Sarti C, Tuomilehto J, Vanhanen H, Webster J, Yodfat Y, Birkenhäger WH, Systolic Hypertension in Europe Investigators (2002). The prevention of dementia with antihypertensive treatment: new evidence from the systolic hypertension in Europe (Syst-Eur) study. Arch Intern Med.

[60] Coca A, Monteagudo E, Doménech M, Camafort M, Sierra C (2016). Can the treatment of hypertension in the middle-aged prevent dementia in the elderly?. High Blood Press Cardiovasc Prev.

[61] Levi Marpillat N, Macquin-Mavier I, Tropeano AI, Bachoud-Levi AC, Maison P (2013). Antihypertensive classes, cognitive decline and incidence of dementia: a network meta-analysis. J Hypertens.

[62] Kherada N, Heimowitz T, Rosendorff C (2015). Antihypertensive therapies and cognitive function: a review. Curr Hypertens Rep.

[63] Yagi S, Akaike M, Ise T, Ueda Y, Iwase T, Sata M (2013). Renin-angiotensin-aldosterone system has a pivotal role in cognitive impairment. Hypertens Res.

[64] Kehoe PG, Wilcock GK (2007). Is inhibition of the renin-angiotensin system a new treatment option for Alzheimer’s disease?. Lancet Neurol.

[65] Savaskan E, Hock C, Olivieri G, Bruttel S, Rosenberg C, Hulette C, Müller-Spahn F (2001). Cortical alterations of angiotensin converting enzyme, angiotensin II and AT1 receptor in Alzheimer’s dementia. Neurobiol Aging.

[66] Bosch J, Yusuf S, Pogue J, Sleight P, Lonn E, Rangoonwala B, Davies R, Ostergren J, Probstfield J, HOPE Investigators. Heart outcomes prevention evaluation (2002). Use of ramipril in preventing stroke: double blind randomised trial. BMJ.

[67] Ohrui T, Tomita N, Sato-Nakagawa T, Matsui T, Maruyama M, Niwa K, Arai H, Sasaki H (2004). Effects of brain-penetrating ACE inhibitors on Alzheimer disease progression. Neurology.

[68] Lithell H, Hansson L, Skoog I, Elmfeldt D, Hofman A, Olofsson B, Trenkwalder P, Zanchetti A, SCOPE Study Group (2003). The Study on Cognition and Prognosis in the Elderly (SCOPE): principal results of a randomized double-blind hypertension trial. J Hypertens.

[69] Li NC, Lee A, Whitmer RA, Kivipelto M, Lawler E, Kazis LE, Wolozin B (2010). Use of angiotensin receptor blockers and risk of dementia in a predominantly male population: prospective cohort analysis. BMJ.

[70] Fogari R, Mugellini A, Zoppi A, Lazzari P, Destro M, Rinaldi A, Preti P (2006). Effect of telmisartan/hydrochlorothiazide vs lisinopril/hydrochlorothiazide combination on ambulatory blood pressure and cognitive function in elderly hypertensive patients. J Hum Hypertens.

[71] Yagi S, Akaike M, Aihara K, Iwase T, Yoshida S, Sumitomo-Ueda Y, Ikeda Y, Ishikawa K, Matsumoto T, Sata M (2011). High plasma aldosterone concentration is a novel risk factor of cognitive impairment in patients with hypertension. Hypertens Res.

[72] Khachaturian AS, Zandi PP, Lyketsos CG, Hayden KM, Skoog I, Norton MC, Tschanz JT, Mayer LS, Welsh-Bohmer KA, Breitner JC (2006). Antihypertensive medication use and incident Alzheimer disease: the Cache County Study. Arch Neurol.

[73] Sze KH, Sim TC, Wong E, Cheng S, Woo J (1998). Effect of nimodipine on memory after cerebral infarction. Acta Neurol Scand.

[74] Hanon O, Pequignot R, Seux ML, Lenoir H, Bune A, Rigaud AS, Forette F, Girerd X (2006). Relationship between antihypertensive drug therapy and cognitive function in elderly hypertensive patients with memory complaints. J Hypertens.

[75] Applegate WB, Pressel S, Wittes J, Luhr J, Shekelle RB, Camel GH, Greenlick MR, Hadley E, Moye L, Perry HM (1994). Impact of the treatment of isolated systolic hypertension on behavioral variables. Results from the systolic hypertension in the elderly program. Arch Intern Med.

[76] Yasar S, Xia J, Yao W, Furberg CD, Xue QL, Mercado CI, Fitzpatrick AL, Fried LP, Kawas CH, Sink KM, Williamson JD, DeKosky ST, Carlson MC, Ginkgo Evaluation of Memory (GEM) Study Investigators (2013). Antihypertensive drugs decrease risk of Alzheimer disease: Ginkgo Evaluation of Memory Study. Neurology.

[77] Gelber RP, Ross GW, Petrovitch H, Masaki KH, Launer LJ, White LR (2013). Antihypertensive medication use and risk of cognitive impairment: the Honolulu-Asia Aging Study. Neurology.

[78] Prince MJ, Bird AS, Blizard RA, Mann AH (1996). Is the cognitive function of older patients affected by antihypertensive treatment? Results from 54 months of the Medical Research Council’s trial of hypertension in older adults. BMJ.

[79] Benetos A, Bulpitt CJ, Petrovic M, Ungar A, Agabiti Rosei E, Cherubini A, Redon J, Grodzicki T, Dominiczak A, Strandberg T, Mancia G (2016). An expert opinion from the european society of hypertension-European union geriatric medicine society working group on the management of hypertension in very old, frail subjects. Hypertension.

